# Does Medicare Coverage Improve Cancer Detection and Mortality Outcomes?

**DOI:** 10.1002/pam.22199

**Published:** 2020-01-12

**Authors:** Rebecca M. Myerson, Reginald D. Tucker‐Seeley, Dana P. Goldman, Darius N. Lakdawalla

## Abstract

Medicare is a large government health insurance program in the United States that covers about 60 million people. This paper analyzes the effects of Medicare insurance on health for a group of people in urgent need of medical care: people with cancer. We used a regression discontinuity design to assess impacts of near‐universal Medicare insurance at age 65 on cancer detection and outcomes, using population‐based cancer registries and vital statistics data. Our analysis focused on the three tumor sites for which screening is recommended both before and after age 65: breast, colorectal, and lung cancer. At age 65, cancer detection increased by 72 per 100,000 population among women and 33 per 100,000 population among men; cancer mortality also decreased by nine per 100,000 population for women but did not significantly change for men. In a placebo check, we found no comparable changes at age 65 in Canada. This study provides the first evidence to our knowledge that near‐universal access to Medicare at age 65 is associated with improvements in population‐level cancer mortality.

## INTRODUCTION

Medicare is a large government health insurance program in the United States that covered an estimated 60 million people per month in 2019 (Centers for Medicare and Medicaid Services, 2019). Medicare's beneficial impacts on patients’ financial outcomes are well established (Barcellos & Jacobson, 2015; Finkelstein & McKnight, 2008). However, in the overall population, Medicare appears to have only modest effects on mortality. Although mortality for hospitalized patients is lower for those with Medicare insurance, multiple studies have found no detectable effect of Medicare on population‐level mortality (Finkelstein & McKnight, 2008; Polsky et al., 2009). Some have concluded that Medicare has little impact on mortality, while others have noted the difficulty of deriving reliable empirical inferences on this question (Black et al., 2017; Kronick, 2009; McWilliams et al., 2010; Polsky et al., 2010). We hypothesize that small average effects could mask important heterogeneity in the effect of Medicare on population health. We study the health effects of Medicare for a group of people for whom access to health insurance could have important short‐run impacts on health outcomes—people with cancer.

In the United States (U.S.), cancer is the second leading cause of death nationwide and the first leading cause of death in certain states, and people over age 65 account for 70 percent of all cancer deaths (Harding et al., 2018; National Cancer Institute Surveillance, Epidemiology, and End Results Program, 2019; White et al., 2014). Timely detection of certain cancers can improve treatment outcomes and reduce mortality risk (Humphrey et al., 2002; Maciosek et al., 2006; Mandelblatt et al., 2009; Moyer & U.S. Preventive Services Task Force, 2012, 2014; Nelson et al., 2009; Pignone et al., 2002; Siu & U.S. Preventive Services Task Force, 2016; U.S. Preventive Services Task Force, 2008). The Institute of Medicine noted that uninsured people experience longer delays in diagnosis and worse health outcomes than patients with private insurance (Institute of Medicine [U.S.] Committee on Health Insurance Status and Its Consequences, 2009). Yet, economists have questioned whether such associations represent a causal effect of insurance or confounding factors (Levy & Meltzer, 2008). Because approximately half of newly diagnosed cancer patients are over age 65, Medicare is the largest payer of cancer care in the United States; thus, resolving uncertainty about the effects of Medicare insurance is crucial for ongoing public policy discussions (National Cancer Institute Surveillance, Epidemiology, and End Results Program, 2019).

The goal of this study was to determine the impact of Medicare's nearly universal coverage at age 65 on cancer detection and cancer mortality. Because cancer detection and treatment are considered health‐improving for some cancers but not others, we focused on the tumor sites with A‐ and B‐grade screening recommendations from United States Preventive Services Task Force, which indicate an evidence‐based recommendation of the service, for people both below and above age 65 (U.S. Preventive Services Task Force, 2018). We therefore analyzed breast, colorectal, and lung cancer, tumor sites for which screening is recommended both above and below age 65. We used the most recent 15 years of data on cancers reported to population‐based cancer registries, survey data, and vital statistics databases across the United States.

We found that concurrent with near‐universal Medicare coverage at age 65, cancer detection increased by 72 per 100,000 population among women and 33 per 100,000 population among men; cancer mortality also decreased by nine per 100,000 population for women but did not significantly change for men. Multiple checks, including comparison with data from Canada as a placebo check, suggested the robustness of findings. Increases in access to health care and cancer screenings at age 65 suggested possible pathways underlying these changes in cancer detection and outcomes. In summary, this study provides the first evidence to our knowledge that near‐universal access to Medicare at age 65 is associated with improvements in population‐level cancer mortality, and provides new evidence on the differences in the impact of health insurance by gender.

### Comparison with the Literature

The association between insurance and cancer outcomes varies by source of insurance coverage, with some publicly insured patients faring no better than the uninsured (Ellis et al., 2018; Halpern et al., 2008; Niu et al., 2013; Ward et al., 2010). To understand which of these associations represent causal effects, researchers can study insurance experiments or leverage a policy change as a natural experiment. Many such studies have focused on insurance expansions among the non‐elderly, i.e., expansions of Medicaid or private insurance. While some studies found that access to insurance increased cancer screening, others found that the impact of insurance on cancer screening and detection varied by tumor site or the length of follow‐up after a policy change (Han et al., 2016; Kolstad & Kowalski, 2012; Robbins et al., 2015; Sabik & Bradley, 2016; Soni et al., 2018). The Oregon Health Insurance Experiment, a randomized expansion of Medicaid insurance, found that insurance increased cancer screening, but cancer detection and outcomes were not assessed (Baicker et al., 2013; Wright et al., 2016).

Given the lower incidence of cancer and lower rates of cancer mortality among the non‐elderly, these studies of Medicaid or private insurance expansions in the non‐elderly face limited statistical power to detect effects on cancer mortality outcomes (National Cancer Institute Surveillance, Epidemiology, and End Results Program, 2019; White et al., 2014). In contrast, by studying an elderly population, our research has enhanced statistical power to detect changes in population‐level cancer mortality.

Prior research has linked Medicare to improvements in self‐reported health, survival after acute care hospital visits, racial/ethnic gaps in measures of cardiovascular health, and access to inpatient and outpatient care (Card, Dobkin, & Maestas, 2008; Card et al., 2009; Decker, 2005; McWilliams et al., 2009; McWilliams et al., 2007). The results of studies linking Medicare and mortality are more mixed, with population‐level studies often finding no effect (Finkelstein & McKnight, 2008; Polsky et al., 2009). Studies on the impacts of Medicare Part D found an impact on cardiovascular mortality but not cancer mortality; however, as the authors note, the majority of cancer treatments were already covered by Medicare Part B prior to the onset of Part D (Dunn & Shapiro, 2017; Huh & Reif, 2017). Studies of the mortality effects of health insurance expansions among non‐elderly adults have found mixed effects; a randomized trial found no impact on mortality, though confidence intervals were large (Black et al., 2017; Finkelstein et al., 2011; Sommers, Baicker, & Epstein, 2012; Woolhandler & Himmelstein, 2017). Yet, we hypothesize that these small or null average relationships between health insurance and mortality could mask important heterogeneity, including significant health effects for policy‐relevant groups such as patients with cancer.

Researchers have also compared cancer survival outcomes across different Medicare plans, and examined the relationship between cancer diagnosis and Medicare plan selection. Medicare beneficiaries can choose to receive their benefits via traditional Medicare (the publicly administered Medicare plan) or Medicare Advantage (a Medicare plan paid for by the federal government but administered by a private company). After a cancer diagnosis, patients become less likely to leave traditional Medicare for a private Medicare Advantage plan, and become more likely to switch from a private Medicare Advantage plan to traditional Medicare (Lissenden, 2019). The literature comparing cancer survival across traditional Medicare and private Medicare Advantage plans has found mixed results depending on the year of the data, tumor site of interest, and controls used to address patients’ self‐selection into plans (Lee‐Feldstein, Feldstein, & Buchmueller, 2002; Lee‐Feldstein et al., 2001; Lissenden, 2019; Merrill et al., 1999; Potosky et al., 1999; Potosky et al., 1997).

Our research question is distinct from this literature, in that we examine the effect of access to the full suite of publicly funded Medicare plans, including both publicly and privately administered plans, rather than the impact of private provision of some Medicare plans. Additionally, these previous analyses focused on post‐diagnosis survival rather than cancer mortality, our outcome of interest. As will be discussed further below, post‐diagnosis survival measures are subject to diagnosis‐related biases: when a disease is detected earlier, the patient will appear to survive longer with the disease even if early detection did not actually extend his or her life.

We are not aware of any prior study of the effect of Medicare coverage on population‐level cancer mortality. The most closely related study to ours focused on the impact of Medicare on post‐detection survival, a different outcome from the one we study, using data from a different time period. Decker examined the impact of Medicare on breast cancer detection, and survival after breast cancer detection (Decker, 2005). Almost all adults in the United States become automatically eligible for Medicare coverage at age 65 (Card et al., 2008). Exploiting this change as a natural experiment, Decker used data on older adults and found that access to Medicare coverage produced small increases in detection of early‐stage breast cancer and post‐detection survival. Decker's study used data from 1980 to 2001. However, since the 1980s and 1990s, changes have occurred that could alter the impact of Medicare on breast cancer detection and outcomes, including new treatments, changes in screening guidelines, changes in the Medicare program, and increases in prevalence of obesity which is associated with breast cancer risk and outcomes (American Cancer Society, 2018; De Pergola & Silvestris, 2013; Picon‐Ruiz et al., 2017; Renehan et al., 2008). Additionally, although the Decker study examined breast cancer, treatment data from Medicare claims suggest that effects may vary by tumor site (Huesch & Ong, 2016a, 2016b).

Importantly, the outcome of survival after cancer detection employed in these prior studies may be subject to diagnosis bias (Feinleib & Zelen, 1969; Lakdawalla et al., 2010; Pinsky, 2015). Diagnosis bias includes lead and length time bias, which can be explained as follows. First, when people are diagnosed with cancer earlier, they may appear to survive longer after detection simply due to becoming classified as a cancer patient earlier—i.e., “lead‐time bias.” Second, the additional tumors detected might be so slow‐growing that they would never have killed the patient if left undetected, resulting in overdiagnosis and overtreatment—i.e., “length bias” (Diederich, 2011; Duffy et al., 2008; Morrison, 1982).

For both these reasons, expansions in cancer detection may improve post‐diagnosis survival even when they do not actually improve health or save lives (Ahn, Kim, & Welch, 2014; Shwartz, 1980). Therefore, changes in post‐diagnosis survival for cancer patients after changes in screening and detection should be interpreted with caution (Barratt, Bell, & Jacklyn, 2018; Croswell, Ransohoff, & Kramer, 2010; Grubbs et al., 2013).

Our study addresses the issues of diagnosis bias by analyzing disease‐specific mortality rates on the population level. Diagnosis bias changes when people are diagnosed, but not when they die. As a result, analyzing population‐level disease‐specific mortality rates is considered a best practice to address diagnosis bias (Croswell et al., 2010; Duffy et al., 2008; Morrison, 1982; Pinsky, 2015).

A final contribution of our study to the literature is our analysis by gender. Prior studies of the impacts of Medicare rarely stratified the data by gender except when studying gender‐specific health care such as mammography (Barcellos & Jacobson, 2015; Card et al., 2008, 2009; Decker, 2005; Dunn & Shapiro, 2017; Huh & Reif, 2017; McWilliams et al., 2003). Yet, it is plausible that gender could play an important role in determining the impact of insurance on health. Several studies have found women to be more likely than men to use preventive health care, and less likely than men to delay seeking needed health care (Bertakis et al., 2000; Galdas, Cheater, & Marshall, 2005; Pinkhasov et al., 2010; Springer & Mouzon, 2011; Vaidya, Partha, & Karmakar, 2012). Gaps by gender in socioeconomic resources could also play a role, as the impact of Medicare could be larger among patients with less‐generous prior insurance coverage and fewer financial resources (Blau & Kahn, 1992; Card et al., 2008; Ruel & Hauser, 2013). Gender differences in the impact of insurance on health have received relatively little attention in the prior literature.

In summary, the impact of Medicare insurance on cancer mortality outcomes is not known. Given that the Medicare population is projected to increase from 54 million in 2015 to 80 million by 2030 and that the older adults served by Medicare will account for 70 percent of cancer patients by 2030, understanding the influence of Medicare coverage on these outcomes is warranted (Medicare Payment Advisory Commission, 2015; Smith et al., 2009).

## METHODS

We employed a regression discontinuity research design. This design assessed the impact of near‐universal Medicare coverage on cancer detection and outcomes at age 65 by comparing data from people aged 65 or slightly older with data from people slightly younger than age 65 (Imbens & Lemieux, 2008). Regression discontinuity designs have been used in prior studies of the impact of Medicare insurance coverage on patient outcomes (Barcellos & Jacobson, 2015; Card et al., 2008; Finkelstein & McKnight, 2008). We use recommended inference practices for regression discontinuity designs (Kolesár & Rothe, 2018). Additional details are provided below.

### Data

We extracted data from multiple sources on cancer detection and mortality, as well as cancer screening, insurance coverage, and access to care in the United States on the population level by age. For use in a placebo test, we additionally extracted data on cancer detection and outcomes just before and after age 65 in Canada, a country without comparable changes in eligibility for public health insurance at age 65.

#### Population‐Level Cancer Mortality

Our primary outcomes of interest were breast, colorectal, and lung cancer mortality per 100,000 population. We used vital statistics data from 2001 through 2015 compiled by the Centers for Disease Control and Prevention, and used the ICD‐10‐based 113 cause list to identify deaths attributed to malignant breast, colorectal, and lung cancer (Centers for Disease Control and Prevention, 2018; Centers for Disease Control and Prevention [CDC] National Death Index [NDI], 2019). Data were tabulated by the location of the cancer, single year of age at death, gender, race (Black vs. non‐Black), and year of death. (Tabulating by state of residence would lead some data to be masked due to low counts. The decision to not tabulate by state is not essential to our findings.) For our main specification that included people aged 59 to 71, this yielded 1.2 million cancer‐related deaths over 1.3 billion person‐years at risk.

#### Cancer Detection

We extracted data on population‐level cancer detection from the Surveillance, Epidemiology, and End Results (SEER) program database from 2001 to 2015, the most recent data available. SEER collects information from population‐based cancer registries covering one‐quarter of the United States population (SEER, 2017). These data include information on patient characteristics and characteristics of the tumor at the time of detection. The SEER data also include information on survival after cancer detection. However, because the data on years of survival after cancer detection are subject to diagnosis bias, we analyzed population‐level cancer mortality rates instead.

Our sample included all cases of breast, colorectal, and lung cancer among people in our age range of interest. Our main specification included people aged 59 to 71, i.e., about 750,000 tumors diagnosed from 136 million person‐years at risk. Data on detected cancers and at‐risk population were tabulated by year, SEER cancer registry, single year of age, gender, and race (Black vs. non‐Black), yielding 13,650 rows of data in our main specification.

Our outcomes of interest from these data were total, early‐stage, and late‐stage cancer detection for breast, colorectal, and lung cancer per 100,000 population. Early‐stage cancer included in situ, localized, or regional by direct extension in the SEER classification. Late‐stage cancer included regional with only lymph node involved, regional with lymph nodes involved and by direct extension, regional not otherwise specified, or distant. Cancers without a stage classification were still included in the analysis of total cancers detected.

#### Comparator Data from Canada on Cancer Detection and Cancer Mortality

In contrast to the abrupt changes in health insurance options at age 65 in the United States, there are no abrupt changes in health insurance options at age 65 in Canada. Therefore, in a placebo check, we extracted data on cancer detection and cancer mortality in Canada over 2001 to 2015 from publicly available vital statistics data from Statistics Canada. Statistics Canada releases data for these outcomes by five‐year age bins. Data on cancer deaths from Statistics Canada were not available by tumor site, and therefore we extracted data for all tumor sites combined. In contrast, data on cancer detection from Statistics Canada were available by tumor site, and therefore we extracted data for our three tumor sites of interest. When comparing data from the United States and Canada, we aggregated the United States data to the same level of aggregation as the Canadian data.

#### Insurance Coverage, Access to Health Care, Cancer Screening, and Other Changes at Age 65

To examine potential mechanisms underlying the changes in cancer detection and outcomes, we extracted information on health insurance coverage, access to health care, use of cancer screening, and economic changes relevant to health. Our data source for these variables was the 2001 to 2015 Behavioral Risk Factor Surveillance System (BRFSS) (Centers for Disease Control and Prevention [CDC] Behavioral Risk Factor Surveillance System [BRFSS], 2018). The BRFSS is a repeated cross‐sectional survey that provides nationally representative annual estimates of demographic, economic, and health‐related variables.

Our outcomes of interest from the BRFSS included health insurance coverage, whether the respondent reported having at least one personal doctor or health care provider, whether the respondent reported having a routine checkup in the past 12 months, and whether there was a time in the past 12 months when the respondent needed to see a doctor but could not because of cost. We also tracked receipt of cancer screenings during the past 12 months. Respondents were considered as having been screened for breast cancer during the past 12 months if they reported having a clinical breast exam or a mammography and were considered as having been screened for colorectal cancer during the past 12 months if they reported having a blood stool test, a colonoscopy, or a sigmoidoscopy. We are not aware of any nationally representative data measuring lung cancer screening consistently over 2001 to 2015, and therefore we were unable to assess changes in lung cancer screening at age 65 during our time period of interest. We extracted detailed data on respondent race, age, gender, state of residence, and year of the interview as covariates to use in multivariate modeling. Finally, for use in balance checks, we extracted data on retirement, employment, veteran status, and education.

### Research Design

We used a regression discontinuity (RD) research design to examine the impact of near‐universal Medicare eligibility at age 65 on cancer detection and outcomes. Regression discontinuity designs are frequently used to analyze policies that cause a sudden change in a treatment of interest that cannot be easily manipulated by patients or providers. Birthweight criteria for neonatal intensive care is one example; intensive care is recommended for infants under 1500 grams. Comparing 1502‐gram infants to 1498‐gram infants illustrates how intensive care influences outcomes, since the four‐gram difference is not otherwise likely to materially influence outcomes (Almond et al., 2010). Time of day is another example, since some hospital patients lose insurance coverage precisely at midnight. Comparing data just before or after midnight identifies how patient insurance status influences hospital treatment decisions (Almond & Doyle, 2011). We exploited patient age as a source of change in insurance coverage: Medicare coverage is nearly universally available at age 65, but not one day before it. The key assumption in our analysis was that outcomes would have continued along a smooth trend at age 65 in the absence of the Medicare program, but Medicare creates a break in that trend. Because smooth trends by age are accounted for in the analysis, it would not invalidate our research design if cancer mortality were to increase with age overall.

Our research design followed previous studies that used regression discontinuity models to estimate the impact of Medicare insurance (Barcellos & Jacobson, 2015; Card et al., 2009; Decker, 2005). We restricted the data to a small window around the Medicare eligibility threshold (age 65) to compare outcomes for people just over age 65 to people just under age 65. To select the size of this window, we used the *rdbwselect* Stata command, which identifies the bandwidth with the best mean squared error for a given application and data set (Calonico, Cattaneo, & Farrell, 2018; Calonico et al., 2017). This procedure yielded an optimal bandwidth of six years in our application. We subsequently assessed the robustness of our findings to changes in the bandwidth.

To estimate the size of the discontinuities in cancer detection and survival at age 65, we employed standard methods for analysis of a regression discontinuity analysis (Imbens & Lemieux, 2008; Lee & Lemieux, 2010), and used recommended inference methods for discretely measured running variables (Kolesár & Rothe, 2018). We estimated the following model for people of age *a*, gender *g*, and race *r*, living in state *s* in year *t*:

$$Y_{agrst}∼f\left(\delta_{0}+\delta_{1}\left(a\geq 65\right)+\delta_{2}\left(a\geq 65\right)p\left(a\right)+\delta_{3}\left(a<65\right)p\left(a\right)+X\beta +\gamma_{t}+\varphi_{s}\right).$$

$Y_{agrst}$ indicates the outcomes analyzed, such as cancers detected or cancer mortality per 100,000 population. The indicator variable (a≥65) indicates age groups who have reached the age cutoff for Medicare (that is, strictly over age 64). This model adjusted for patient gender and race (covariates in vector *X*), as well as year and state fixed‐effects ($\gamma_{t}\;\mathrm{and}\;\varphi_{s}$). The effects of age were allowed to vary above vs. below the cutoff using a polynomial in age centered at age 65 (p(a)). Our main specification used a quadratic polynomial, but we present results from linear and cubic functions in robustness checks. δ_1_ is the coefficient of interest, capturing the additional change in the outcome of interest at age 65. We used Eicker‐Huber‐White (EHW) heteroscedasticity‐robust standard errors for inference, based on the superior coverage properties of these standard errors compared to clustering standard errors by the running variable for small to moderate size bandwidths such as ours (Kolesár & Rothe, 2018). We assessed variation in the findings by race or tumor site, and before versus after the introduction of Medicare Part D in 2006, by stratifying the data.

The functional form of the models used varied by the outcome analyzed. We used negative binomial models to assess changes in rates such as cancer detection per population or cancer deaths per population and used logit models to assess changes in binary outcomes such as health insurance coverage or cancer screening during the past year. We presented average marginal effects capturing the additional change at age 65 as our quantity of interest from each of these models.

#### Sensitivity Checks

We assessed the assumption that outcomes would have remained smooth at age 65 in the absence of the Medicare program in two ways. First, we examined changes in other socioeconomic variables, such as retirement at age 65, using the BRFSS data, following previous studies that studied Medicare using a regression discontinuity design (Barcellos & Jacobson, 2015; Card et al., 2008). Second, we examined changes in our outcomes of interest at age 65 using data from Canada, a country that does not have a change in public insurance coverage at age 65.

We further assessed the robustness of findings by changing the model specifications. First, we assessed the sensitivity of our results to our chosen age window by reestimating our models on a narrower sample of people aged 61 to 69, and a broader sample of people aged 57 to 73. Second, we changed the order of the polynomial used to adjust for the aging process to a linear or cubic polynomial. Third, we used an alternate modeling approach, implementing a quadratic polynomial estimator with robust bias‐corrected confidence intervals under second‐order Holder smoothness class using the *LPPHonest* function from the *RDHonest* package in R (Kolesár & Rothe, 2018). This method used a triangular kernel to place a higher weight on observations closer to age 65.

All analyses used data from 2001 to 2015. Analyses were conducted using Stata MP, version 14.1, and R, version 3.6.0. We assessed statistical significance at the 0.05 level using two‐sided tests and calculated 95 percent confidence intervals for each quantity of interest.

## RESULTS

**Figure 2:**
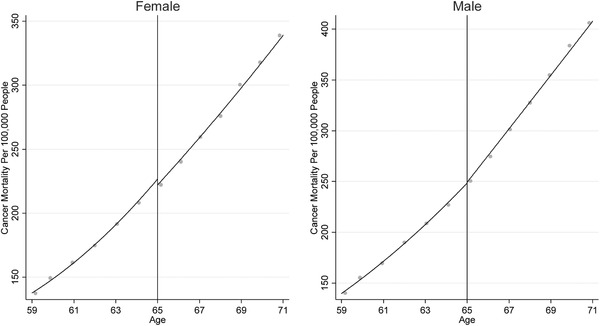
Population‐Based Cancer Mortality in the United States 2001 to 2015: Vital Statistics Data. *Notes*: This graph shows trends in population‐level cancer mortality from our tumor sites of interest just above and below age 65, the age of near‐universal health insurance coverage via Medicare. The x‐axis is age; and the y‐axis is cancer mortality per 100,000 population. The graph includes quadratic regression lines, estimated separately below vs. above age 65.

Figures 1 and 2 depict cancer detection rates and cancer mortality rates in the United States over 2001 to 2015, just before and after near‐universal eligibility for Medicare at age 65. These data have three notable characteristics. First, cancer detection and cancer mortality increase with age in general, reflecting the overall aging process. Second, there are visible increases in cancer detection at age 65 for both men and women. Third, cancer mortality appears to decline—i.e., increase by less than would be expected based on prior trends—at age 65 among women, whereas there is no break in trend at age 65 among men.

Figure 1Cancer Detection Per 100,000 Population Among Patients Just Above and Below Age 65 in the United States Over 2001 to 2015: SEER Data.
*Notes*: This figure depicts trends in detection of cancers for our tumor sites of interest just above and below age 65, the age of near‐universal health insurance coverage via Medicare. The x‐axis is age at diagnosis; and the y‐axis is cancers detected per 100,000 population. The graphs include quadratic regression lines, estimated separately below vs. above age 65.

**Figure:**
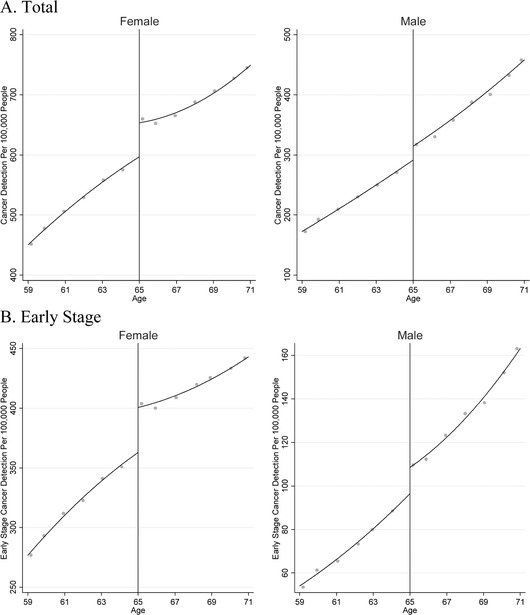

**Figure:**
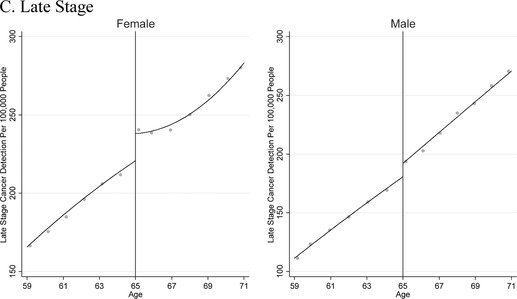

Table 1 shows our regression discontinuity estimates of the change in cancer detection and cancer mortality at age 65, after adjusting for aging and other factors. The first two columns of Table 1 present the data when men and women were pooled together. Cancer detection increased by 50 per 100,000 population (95 percent confidence interval 31 to 69 per 100,000 population), and early‐stage cancers accounted for much of the increase (33 per 100,000 population, 95 percent confidence interval 21 to 44 per 100,000 population). This represented a 10 percent decrease in cancer detection and a 12 percent increase in early‐stage cancer detection among 65‐year‐olds compared to people aged 63 to 64, the “untreated” group in our analysis. When data from men and women were pooled together, there was not a statistically significant change in cancer mortality, and findings were similar for Black and non‐Black patients. The changes in cancer detection and mortality were similar before and after the onset of Medicare Part D in 2006.

**Table 1:** Impact of near‐universal access to Medicare on cancer detection and cancer mortality in the United States 2001 to 2015: SEER and Vital Statistics Data

A. Cancer Detection per 100,000 Population						
	All		Women		Men	
	Age 63–64	RD at Age 65	Age 63–64	RD at Age 65	Age 63–64	RD at Age 65
Total	507.3	50.05***	683.7	71.58***	312.8	33.19***
		(30.88 to 69.22)		(51.49 to 91.67)		(15.81 to 50.57)
*By Stage*						
Early Stage	267	32.91***	417.4	47.45***	101.2	17.39***
		(21.47 to 44.35)		(33.13 to 61.76)		(8.03 to 26.74)
Late Stage	226	19.36***	252.1	22.28***	197.4	17.90***
		(9.42 to 29.30)		(10.88 to 33.68)		(4.76 to 31.03)
*By Tumor Site*						
Breast		N/A	459.2	50.05***		N/A
				(35.35 to 64.75)		
Colorectal	82.5	16.34***	69.62	13.83***	96.61	18.79***
		(10.37 to 22.31)		(6.40 to 21.25)		(9.47 to 28.11)
Lung	182.3	10.79**	154.9	7.47	212.5	15.09**
		(1.545 to 20.04)		(−2.475 to 17.40)		(0.733 to 29.44)
*By Race*						
Black	609.7	42.60**	725.1	77.28***	465.9	9.60
		(6.55 to 78.65)		(28.21 to 126.3)		(−34.91 to 54.11)
Non‐Black	495.9	52.18***	678.8	70.87***	297	34.15***
		(33.00 to 71.36)		(49.77 to 91.98)		(18.14 to 50.16)
*By Time*						
Prior to 2006	561.1	39.81**	561.1	40.77**	726.1	52.22***
		(4.95 to 74.67)		(4.78 to 76.77)		(18.11 – 86.34)
2006 and later	488.4	53.72***	488.4	83.99***	290	25.72***
		(31.78 to 75.65)		(61.21 – 106.8)		(6.31 to 45.12)
Total	208.3	−4.13	199.8	−8.93**	217.7	2.92
		(−14.31 to 6.05)		(−17.39 to –0.46)		(−6.48 to 12.31)
*By Tumor Site*						
Breast		N/A	59.96	0.16		N/A
				(−2.99 to 3.31)		
Colorectal	37.1	0.48	29.25	−0.24	45.7	1.46
		(−1.31 to 2.28)		(−2.37 to 1.89)		(−1.45 to 4.37)
Lung	139.5	−4.26	110.6	−6.64**	171.3	0.87
		(−14.28 to 5.76)		(−12.72 to −0.57)		(−7.52 to 9.26)
*By Race*						
Black	270.0	−8.34	238.4	−20.29***	309.6	9.59
		(−21.15 to 4.48)		(−31.74 to −8.84)		(−6.65 to 25.83)
Non‐Black	201.3	−1.59	195.1	−3.32	208.0	0.38
		(−8.79 to 5.61)		(−11.14 to 4.51)		(−7.90 to 8.66)
*By Time*						
Prior to 2006	208.3	−2.97	234.2	−8.23	266.7	7.13
		(−20.59 to 14.66)		(−19.64 to 3.17)		(−9.27 to 23.53)
2006 and later	208.3	−2.62	187.5	−7.15	200.3	3.50
		(−12.18 to 6.94)		(−16.22 to 1.93)		(−6.48 to 13.46)

*Notes*: The first two columns of each table include findings when all data from cancers with recommended screening are pooled together. The subsequent columns include findings from stratified analyses including only women or men, respectively. Estimated regression discontinuities at age 65 are adjusted for background trends in aging and over time and patient gender and race; cancer detection analyses are additionally adjusted for time‐invariant confounders by state. 95 percent confidence intervals calculated using robust standard errors are in parentheses. *p<0.1; **p<0.05; ***p<0.01.

The remaining columns of Table 1 show the changes at age 65 when the data were stratified by gender. At age 65, cancer detection increased by 72 per 100,000 population among women, or 11 percent, and 33 per 100,000 population among men, or 11 percent (95 percent CI 52 to 92 and 16 to 51, respectively). Cancer mortality declined among women by nine per 100,000 population, or 4.5 percent (95 percent CI zero to 17 per 100,000 population); we did not find a significant change in cancer mortality among men. Breast cancer accounted for the additional tumors detected among women, whereas lung cancer accounted for the decline in cancer mortality when all women were analyzed together. Figures A1 and A2 depict the data stratified by tumor site and gender.1All appendices are available at the end of this article as it appears in JPAM online. Go to the publisher's website and use the search engine to locate the article at http://onlinelibrary.wiley.com.


**Figure A4:**
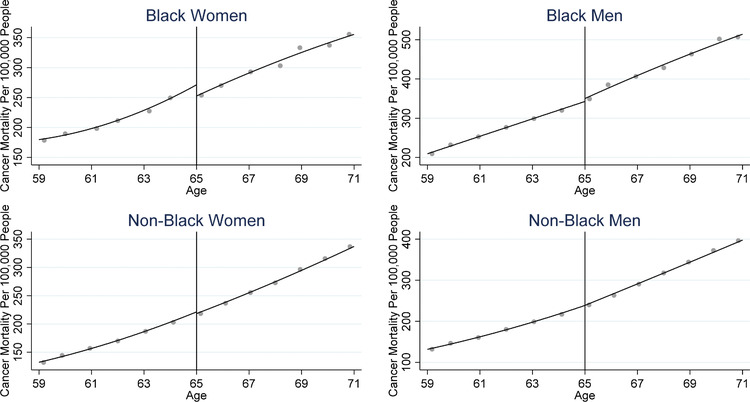
Population‐Based Cancer Mortality in the United States 2001 to 2015: Vital Statistics Data. *Notes*: This figure shows trends in population‐level cancer mortality from our tumor sites of interest just above and below age 65, the age of near‐universal health insurance coverage via Medicare, by gender and race. The x‐axis is age and the y‐axis is cancer mortality per 100,000 population. The graphs include quadratic regression lines, estimated separately below vs. above age 65.

Data stratified by gender and race are shown in Table 1, Table A1, and Figures A3 and A4. Racial disparities in stage of cancer detection and mortality prior to age 65 are notable. At ages 63 to 64, only 55 percent of Black women with cancer had been diagnosed prior to metastasis, compared with 62 percent of non‐Black women. Additionally, at ages 63 to 64, Black women had a cancer mortality rate that was 22 percent higher than non‐Black women. Subsequently, at age 65, Black women experienced a particularly large increase in early‐stage cancer detection (62 per 100,000 population or 15 percent, compared to an increase of 47 per 100,000 population or 11 percent among non‐Black women). Black women also experienced a particularly large decline in cancer mortality at age 65, of 20 per 100,000 population (95 percent confidence interval: nine to 32), or 9 percent. Breast cancer mortality and lung cancer mortality may have both contributed to this mortality decline among Black women, although the changes were only statistically significant at the 10 percent level: declines for breast and lung cancer mortality were seven per 100,000 population and nine per 100,000 population, respectively (95 percent confidence intervals ‐1 to 14 and ‐0 to 19, respectively). In contrast, we found no significant change in cancer detection or mortality among Black men.

**Figure 3:**
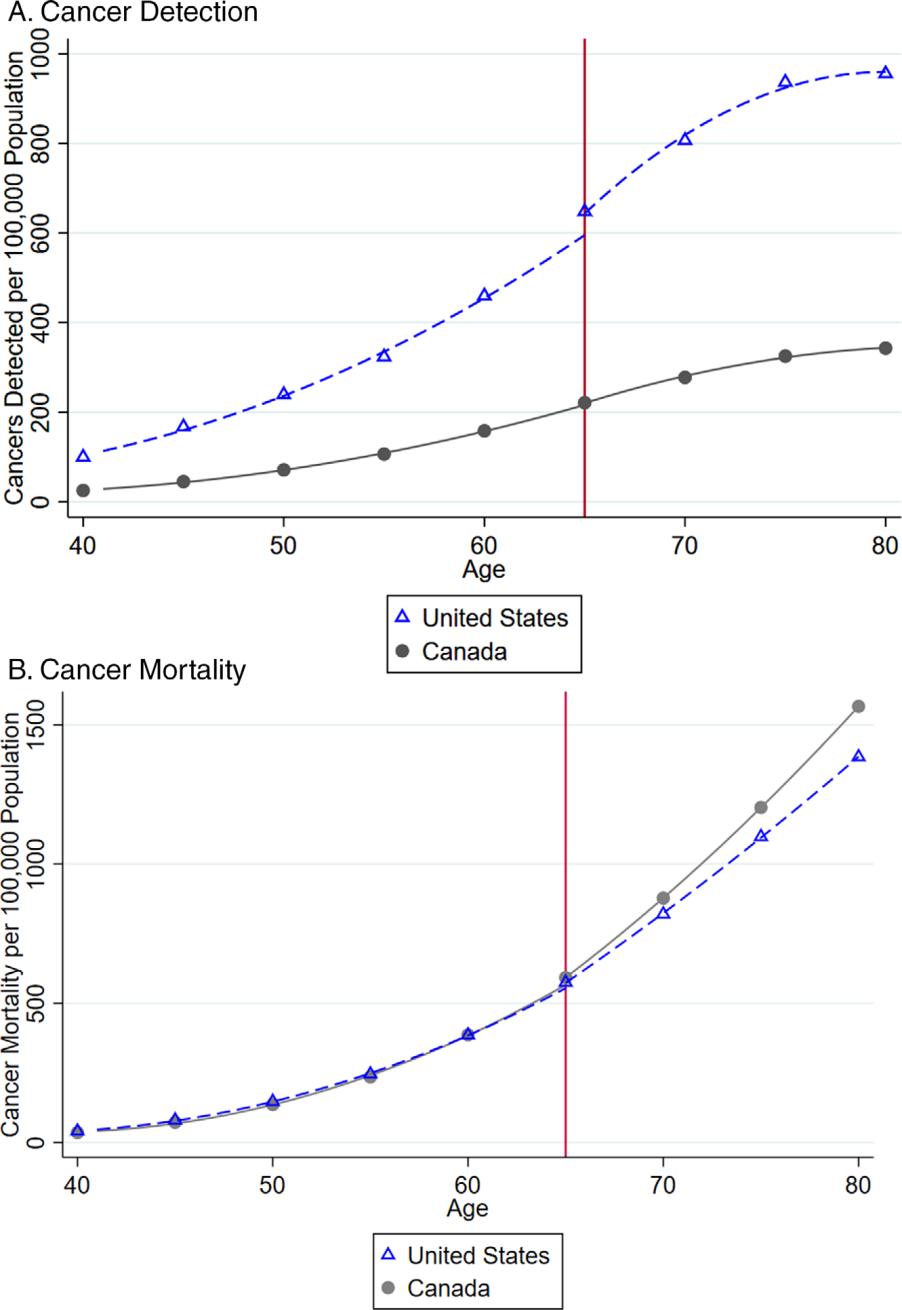
Cancer Detection and Mortality Just Above and Below Age 65 in 2001 to 2015 in the United States vs. Canada. *Notes*: The x‐axis is age at diagnosis and the y‐axis is cancer deaths or cancer detection per 100,000 population. The graphs include quadratic regression lines, estimated separately before and after age 65. The Canadian mortality data are not available by tumor site, and the Canadian detection and mortality data are not available by single year of age. We aggregated the United States data to the same level of aggregation as the Canadian data. [Color figure can be viewed at wileyonlinelibrary.com]

The first identifying assumption of the regression discontinuity design is that cancer detection and outcomes would have remained smooth at age 65 if the onset of Medicare coverage had not occurred. To assess the plausibility of this assumption, we examined data from Canada. Canada does not have a change in access to public health insurance coverage at age 65, given that permanent residents and citizens of any age are provided access to public health insurance under the Canada Health Act. Figure 3 compares data from the United States and Canada. Cancer mortality was nearly identical in the United States and Canada prior to age 65, but at ages 65 and older the outcomes diverged, with the mortality rates ultimately becoming lower in the United States. Additionally, cancer detection remained on a smooth path before and after age 65 in Canada but showed a break in trend at age 65 in the United States.

**Figure A5:**
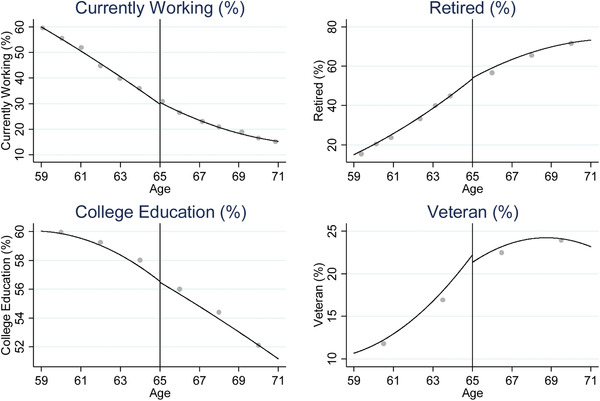
Participant Characteristics Just Above and Below Age 65 in the United States Over 2001 to 2015: BRFSS Data. *Notes*: This figure shows trends in key economic and demographic variables just above and below age 65, the age of near‐universal health insurance coverage via Medicare. The x‐axis is age and the y‐axis is percentage of people with each characteristic of interest. The graphs include quadratic regression lines, estimated separately below vs. above age 65. Analyses used sample weights to reflect the complex sampling design of the BRFSS data.

To further assess the plausibility that our outcomes of interest would have remained smooth at age 65 if the onset of Medicare coverage had not occurred, we assessed trends in economic determinants of health at age 65. Prior studies that also used a regression discontinuity design to analyze Medicare have assessed trends in economic determinants of health at age 65, but these studies used data from earlier time periods (Barcellos & Jacobson, 2015; Card et al., 2008). We performed similar tests using BRFSS data from our time period of interest, 2001 to 2015, and our results were similar to previous studies. See Figure A5 and Table A2. We found no statistically significant discontinuity at age 65 in the proportion of survey respondents who were currently working, who had a college education, or who were veterans. Despite the lack of a change in the proportion of respondents currently working, we found a statistically significant change in the proportion of respondents who considered themselves retired of 1.2 percentage points (95 percent confidence interval 0.0 to 2.5 percentage points). This change in retirement was smaller in magnitude than the changes in health insurance coverage at age 65, as Card and colleagues also found, and could reflect the possibility that when public health insurance is unavailable people may wish to work simply to secure health insurance (Card et al., 2008; Garthwaite, Gross, & Notowidigdo, 2014).

The second identifying assumption of the regression discontinuity design is that people do not manipulate their age in order to gain access to the program. Multiple aspects of the Medicare program and the data used in this paper suggest that this assumption is reasonable. First, people cannot alter their age. Second, people have little ability or incentive to misreport their age in this context we are studying. The Medicare program is administered by the federal government in accordance with official records such as birth certificates, presenting steep obstacles to misrepresenting one's age later in life. Additionally, our main data source for mortality is vital statistics data, gathered from death certificates. The deceased have no ability, and surely no incentive, to misreport their age at death in official records. Nonetheless, if coroners or family members of the deceased misreported age at death by rounding the deceased's age to numbers ending in five or zero, this would result in excess deaths at age 65 and bias our results towards the null.

**Figure 4:**
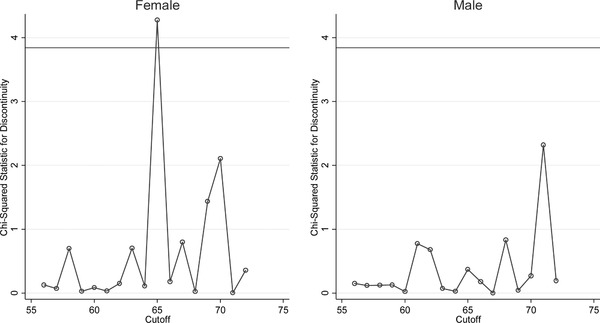
Findings from Chi‐Square Tests for Discontinuity in Population‐Level Cancer Mortality at Age 65 and Other Ages. *Notes*: This figure shows that the largest and most statistically significant discontinuity in cancer mortality per 100,000 population, within the bandwidth we analyze, is at age 65, as expected. The chi‐squared statistics plotted tested the significance of the discontinuity in cancer mortality found when different ages were used as the cutoff in our regression discontinuity analysis. The horizontal line depicts the cutoff for statistical significance at the 0.05 level.

In placebo tests, we did not find comparable changes in population‐level cancer mortality at ages other than 65. See Figure 4. This evidence supports the plausibility that changes at age 65, concurrent with near‐universal eligibility for Medicare, accounted for our findings. Our point estimates were similar when we reestimated our models on a narrower sample of people aged 61 to 69, or a broader sample of people aged 57 to 73; or when we used a linear or cubic polynomial to model the aging process rather than a quadratic polynomial. When we used a less parametric approach, the estimated cancer detection effects became smaller but still statistically significant, and the cancer mortality results became statistically significant for all race and gender groups. See Table A3.2All appendices are available at the end of this article as it appears in JPAM online. Go to the publisher's website and use the search engine to locate the article at http://onlinelibrary.wiley.com.


Improvements in insurance coverage, access to care, and cancer screening at age 65 could help to explain our findings. Therefore, we also examined the changes in these intermediate outcomes at age 65. See Figure 5 and Table 2. These data indicate that insurance coverage rates, affordability of care, and colorectal cancer screening improved for both men and women. Breast cancer screening rates, which were only measured among women, showed a statistically significant increase at age 65. The data also show that women showed statistically significant improvements at age 65 in having a personal doctor and an annual check‐up, whereas men did not. Figure A6 shows the raw data for all these outcomes.

Figure 5Health Insurance Coverage and Access to Care Just Above and Below Age 65 in the United States Over 2001 to 2015: BRFSS Data.
*Notes*: This figure shows trends in population‐level health insurance coverage, access to care, and cancer screening just above and below age 65, the age of near‐universal access to Medicare. The x‐axis is age and the y‐axis is the percentage of people with the characteristic listed on the axis. The graphs include quadratic regression lines, estimated separately below vs. above age 65.

**Figure:**
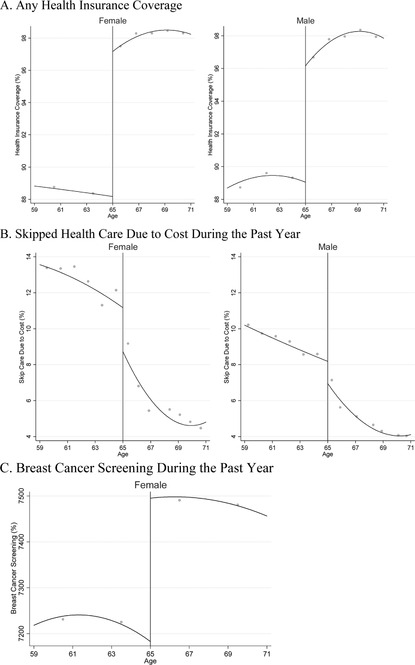

**Figure:**
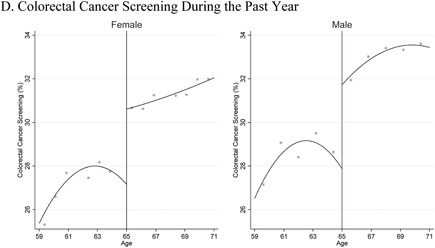

**Table 2:** Impact of near‐universal access to Medicare at age 65 on insurance coverage, access to care, and cancer screening in the United States 2001 to 2015: BRFSS Data

	Women		Men	
	Age 63–64	RD at Age 65	Age 63–64	RD at Age 65
Health Insurance Coverage	0.88	0.045***	0.89	0.043***
		(0.041 to 0.048)		(0.038 to 0.047)
*Access to Care*:				
Personal Doctor	0.92	0.009**	0.89	−0.002
		(0.001 to 0.016)		(−0.014 to 0.010)
Check‐Up	0.80	0.033***	0.78	0.011
		(0.022 to 0.043)		(−0.003 to 0.026)
Not Access Care Due to Cost	0.12	−0.020***	0.09	−0.010**
		(−0.028 to −0.011)		(−0.020 to −0.0002)
*Screenings During Past Year*:				
Breast Cancer	0.74	0.024***	Not reported	Not reported
		(0.007 to 0.041)		
Colorectal Cancer	0.29	0.024**	0.30	0.033***
		(0.004 to 0.043)		(0.008 to 0.057)

*Notes*: This table shows the results of regression discontinuity analyses at age 65, which assessed discontinuities in insurance coverage, access to care, and breast and colorectal cancer screening during the past year. (Lung cancer screening was not measured consistently during our time period of interest.) Estimated regression discontinuities at age 65 adjusted for background trends in aging and over time, time‐invariant confounders by state, and respondent race. Models incorporated sample weights to account for the complex sampling design of the BRFSS data. 95 percent confidence intervals calculated using robust standard errors are in parentheses.

Our regression discontinuity design identified the marginal effects of Medicare at age 65, rather than the full effect of Medicare among adults aged 65 and older. Thus, any cost and benefit calculations stemming from our estimates should be restricted to assessing the cost per cancer death averted at age 65. In a back‐of‐the‐envelope calculation, we estimated Medicare's expenditure on related treatment per breast, colorectal, or lung cancer death averted by Medicare at age 65. Our expenditure estimates included payments by Medicare for patients diagnosed at 65 as well as patients diagnosed before 65 who subsequently survived to age 65. We used estimates from the literature of the per‐patient Medicare expenditures associated with ongoing or initial treatment related to breast, colorectal, and lung cancer (Yabroff et al., 2008). Additional details of the calculation are included in the Appendix. This back‐of‐the‐envelope calculation suggested that Medicare spent about $5 million on breast, colorectal, or lung cancer care for each related cancer death averted by the Medicare program at age 65.

## DISCUSSION

The goal of this study was to estimate the impact of Medicare health insurance coverage at age 65 on cancer detection and population‐level cancer mortality over 2001 to 2015. We are not aware of any previous study of the relationship between Medicare insurance coverage at age 65 and population‐level cancer mortality. We studied all tumor sites for which screening is recommended among older adults, yielding a sample of about 750,000 breast, colorectal, and lung cancer cases diagnosed among patients aged 59 to 71.

Insurance coverage rose to 97 percent at age 65, the age of near‐universal eligibility for Medicare. This nearly universal Medicare coverage increased cancer detection by 50 per 100,000 population, a 10 percent increase compared to people aged 63 to 64; most of the additional tumors detected were early‐stage. These findings are important for population health because prompt detection improves health for the tumor sites we study here, according to systematic reviews by the United States Preventive Services Task Force (Moyer & U.S. Preventive Services Task Force, 2014; Siu & U.S. Preventive Services Task Force, 2016; U.S. Preventive Services Task Force, 2008). Supplemental analyses, including analysis of data from Canada in a placebo check, supported the robustness of our findings.

In vital statistics data, we found that cancer mortality increased by less than expected among women at age 65 by nine per 100,000 population. The lack of significance among men is consistent with the magnitudes and standard errors we estimated. The increases in cancer detection and early‐stage cancer detection at age 65 among men were both less than half the magnitude of the increases at age 65 among women: total cancer detection increased by 33 per 100,000 population among men compared to 72 per 100,000 population among women, and early‐stage cancer detection increased by 17 per 100,000 population among men compared to 47 per 100,000 population among women. If cancer deaths changed proportionately to cancer detection, the implied change in deaths for men (4.1 per 100,000 population) would not be statistically significant based on the calculated standard error for men (4.8 per 100,000 population).

Our findings by race and gender are also notable and contribute to the literature. Black women experienced a particularly large increase in early‐stage cancer detection at age 65, of 62 per 100,000 population or 15 percent, compared to an increase of 47 per 100,000 or 11 percent among non‐Black women. Black women also experienced a statistically significant decline in cancer mortality of 20 per 100,000 population, or 9 percent, at age 65. Unpacking the tumor sites responsible for these improvements, we found possible improvements in breast cancer mortality and lung cancer mortality that reached significance at the 10 percent level. The disproportionate effect of Medicare on cancer detection and mortality among Black women may reflect the higher proportion of cancers detected at a late stage among Black women prior to age 65 and the larger impact of Medicare on cancer screening among Black women (Decker, 2005; Virnig et al., 2009). These findings are particularly important given that Black patients with cancer have worse health outcomes on average than other racial groups (Eley et al., 1994; Howard et al., 1992; Joslyn & West, 2000). Despite these encouraging findings among Black women, we found no significant change in cancer detection or mortality at age 65 among Black men. Efforts to further address disparities in cancer outcomes for Black men should be further explored.

To further clarify the novelty of our analyses and findings, a brief explanation of diagnosis bias and how our analytic strategy addresses such bias may be helpful. Two diagnosis biases applicable in cancer research are lead time bias and length bias, which can be summarized as follows. First, patients whose cancer is diagnosed earlier will appear to live longer after diagnosis even if earlier detection provides no clinical benefit. Second, as detection rates rise, the additional detected cancers may be slow‐progressing cancers that are less deadly. When diagnosis rates rise, these biases may result in spurious improvements in rates of post‐diagnosis survival, simply because the additional diagnosed patients had less severe disease (Barratt et al., 2018; Croswell et al., 2010; Duffy et al., 2008; Feinleib & Zelen, 1969; Pinsky, 2015; Shwartz, 1980).

Our analysis advances beyond the prior literature on the effects of Medicare on cancer survival by addressing diagnosis bias. To avoid the spurious results diagnosis bias could create, we analyze population‐level cancer mortality data from vital statistics records. Analyzing population‐level cancer mortality data is the recommended method to address diagnosis bias because the vital statistics data include deaths by cancer not diagnosed during the patient's lifetime (i.e., cancer diagnosed at autopsy) (Duffy et al., 2008; Morrison, 1982). This makes our approach substantively different from prior analyses of changes in post‐diagnosis survival among Medicare patients (Decker, 2005; Huesch & Ong, 2016a, 2016b; Lissenden, 2019; Merrill et al., 1999; Potosky et al., 1999; Roetzheim et al., 2000). Additional strengths of our study included the use of recent data from an era of rapid advances in cancer treatment, and a large sample size resulting in high statistical power.

Declines in lung cancer mortality accounted for the significant reductions in cancer mortality among women at age 65; we also found declines in breast cancer mortality among Black women that had statistical significance at the 10 percent level. These changes are important for population health because lung and breast cancer are the two leading causes of cancer mortality among women, and account for more than one‐quarter of all productivity costs associated with cancer mortality in the United States (Bradley et al., 2008; Cronin et al., 2018).

Improvements in cancer mortality after the onset of nearly universal Medicare coverage are plausible, given that life‐extending treatments exist but are costly. Randomized trials showed that timely detection and treatment can reduce the risk of lung cancer mortality by 20 percent and breast cancer mortality by 33 percent (National Lung Screening Trial Research Team et al., 2011; Nelson et al., 2016). Treatment can be prohibitively expensive, with an initial year of treatment costing on average $73,000 for lung cancer or $28,000 for breast cancer in 2010 U.S. dollars (Mariotto et al., 2011). The elevated rates of bankruptcy among cancer patients aged 50 to 64 suggest that out‐of‐pocket expenditures associated with treatment may be associated with substantial financial hardship for people lacking Medicare coverage (Ramsey et al., 2013; Zheng et al., 2019).

Our analysis captures a local average treatment effect of the effect of access to Medicare at age 65, i.e., the effect of having access to Medicare compared to lacking access to Medicare at age 65. Given that access to Medicare is nearly universal at age 65 but very limited prior to age 65, our research design compared a “treated” group aged 65 and older to an “untreated” group younger than 65, an approach also used in previous studies (Barcellos & Jacobson, 2015; Decker, 2005; Finkelstein & McKnight, 2008; McWilliams et al., 2009). While access to Medicare may be particularly beneficial for the previously uninsured, the previously insured also benefit. Within a few weeks of becoming eligible for Medicare, there is a sharp increase in the proportion of people with multiple forms of coverage, and the proportion of people with only managed care is reduced by about half (Card et al., 2009). Thus, Medicare provides the already‐insured with access to more generous coverage and a broad network of providers. Generosity of coverage and network breadth are particularly important for cancer patients, who require costly specialty care (Lissenden, 2019; Mariotto et al., 2011; Yabroff et al., 2008).

The increases we find in cancer detection and routine cancer screening capture only some of the possible mechanisms through which Medicare could reduce cancer mortality. Some patients whose lives were extended by an avoided cancer death at age 65 may have been diagnosed prior to age 65. For these patients, Medicare could provide access to needed treatment for an already diagnosed condition. Other patients who became diagnosed and treated upon gaining Medicare coverage may have already had symptoms of cancer. These patients would have become diagnosed by confirmatory testing, rather than by routine cancer screening.

Our findings are consistent with epidemiologic principles on the expected health effects of increased cancer detection. In the cancer epidemiology literature, length bias, overdiagnosis, and overtreatment describe the phenomenon wherein additional diagnosis and treatment of cancer yields diminishing health returns within a population, because the marginal cancer cases are less severe than the previous cases (Ahn et al., 2014; Barratt et al., 2018; Diederich, 2011; Duffy et al., 2008; Morrison, 1982). These concepts map closely to our results by tumor site. Prior to age 65, lung cancer was more likely to be diagnosed at a late stage and had higher post‐diagnosis mortality than the other tumor sites we studied, with fewer than half of lung cancer patients surviving one year after diagnosis. Accordingly, the health returns to increased diagnosis and treatment at age 65 were higher for lung cancer than the other tumor sites. As detection and treatment increase further over time, these epidemiologic principles suggest that health returns to detection and treatment will diminish (Ahn et al., 2014; Esserman, Thompson Jr., & Reid, 2013).

Our findings also reflect principles from health economics about the role access to care plays in determining diagnosis and treatment. Patients lacking access to affordable treatment may delay treatment of diagnosed disease due to cost, or delay diagnosis of a symptomatic condition because a formal diagnosis would provide no actionable next step (Baicker et al., 2013; Brawley & Goldberg, 2012; Oster, Shoulson, & Dorsey, 2013; Wilson, 2016). Thus, patients lacking access to care are particularly likely to be underdiagnosed and undertreated. The diminishing returns to treatment documented in the epidemiological literature, in turn, suggest that improving access for undertreated patients should yield relatively large health gains (Myerson et al., 2018). This prediction maps closely to our findings by race. Prior to age 65, insurance coverage rates were lower and delays in detection and treatment more frequent for Black than for non‐Black patients (Card et al., 2008; Virnig et al., 2009). Subsequently, Black patients experienced larger mortality gains than non‐Black patients upon gaining access to Medicare coverage.

The magnitude of our estimated change in cancer mortality is consistent with previous studies of changes in mortality after health insurance expansions. First, we did not detect any change in the impact of Medicare on cancer mortality after the onset of Medicare Part D. This matches prior research that found no effect of the introduction of Medicare Part D on cancer mortality (Dunn & Shapiro, 2017; Huh & Reif, 2017). As these authors noted, Medicare already covered many cancer treatments prior to the introduction of Part D through Medicare Part B and Medicare Advantage (Part C), so the result is unsurprising. Second, we found a larger impact on cancer mortality among Black women. This matches prior studies that found larger improvements in mortality after Medicaid expansions among racial and ethnic minorities (Sommers et al., 2012). A study of patients hospitalized for non‐deferrable causes such as acute myocardial infarction and stroke found a larger effect of Medicare on mortality than we found, as expected given the authors’ focus on a population with acute illness (Card et al., 2009). Card and colleagues found Medicare was associated with a one percentage point decline in seven‐day mortality (a 20 percent decline), compared to the smaller effect we find of nine cancer deaths averted per 100,000 population among women (a 4.5 percent decline). Also as expected, our estimated effects of Medicare on cancer mortality are smaller than the estimated effect of Medicaid expansions on all‐cause mortality, which was identified by Sommers and colleagues as a reduction by 20 deaths per 100,000 population (a 6 percent decline) (Sommers, 2017; Sommers et al., 2012). Direct comparisons of the results for women and men are not possible given that neither paper stratified the data by gender.

We found no decline in deaths due to breast cancer after aging into Medicare when all women were analyzed together, which may appear to contradict the findings of a previous study that found survival after breast cancer diagnosis improved after aging into Medicare (Decker, 2005). However, Decker reported that the 11 percent improvement in breast cancer survival originally identified became attenuated to 9 percent after adjusting for state at diagnosis. If diagnosis bias contributed to the differences between the findings of this study and ours, then additional controls for tumor severity might have further attenuated the findings. Differences in the data periods used might also account for the differences between the findings. Decker used data from 1980 through 2001, whereas we used data from 2001 through 2015. There was a 39 percent decline in the breast cancer death rate over 1989 to 2015, and detecting further improvements in the cancer death rate may become more challenging as rates fall (DeSantis et al., 2017). In keeping with this possibility, the decline in breast cancer mortality was closer to statistical significance among Black women, who had a higher baseline rate of breast cancer mortality.

Our findings inform ongoing policy discussions about Medicare and the benefits of access to publicly provided insurance. Changes to the Medicare eligibility age have been repeatedly proposed. Informing this policy proposal, our findings suggest that setting the Medicare eligibility age at 65 rather than 66 averted about 1,800 deaths from cancer at age 65 during our sample period (2001 to 2015). While our regression discontinuity design only measured deaths averted at age 65, deaths averted at age 65 are likely a small fraction of the total cancer deaths prevented by Medicare. Indeed, deaths at age 65 accounted for only 4 percent of cancer deaths among adults aged 65 and older in the United States during our time period of interest. The increasing gap in cancer mortality rates between the United States and Canada after age 65 suggests the possibility that effects of the Medicare program on cancer mortality could be larger at older ages. If the effect of Medicare on cancer mortality is larger at older ages, our findings would underestimate the total impact of Medicare on cancer mortality. Regardless, policymakers should take into account the full set of impacts of Medicare, including impacts on financial strain, when making policy decisions (Barcellos & Jacobson, 2015; Finkelstein & McKnight, 2008).

Our study had limitations. First, insurance coverage is not well‐measured among cancer patients in the SEER or vital statistics data. To address this limitation, we used an additional data source to present contextual information about the increase in coverage at age 65, and how changes in coverage translated to changes in access to care. Second, changes in rates are subject to population dynamics. However, our population denominators were updated annually by single year of age, state, gender, and race. Our analysis therefore accounts for any changes in migration or mortality by age, including differential migration or mortality by race or gender. Finally, ours is an observational study; while our analysis of data from Canada can assuage some concerns, we cannot rule out the possibility that changes other than onset of Medicare in the United States at age 65 account for our findings.

In conclusion, access to Medicare insurance was associated with a significant increase in detection of cancers with recommended screening, as well as a decline in mortality from these cancers among women. Our estimates provide new evidence of Medicare's impact on health outcomes for people in need of medical care.

## Calculation of Expenditure per Cancer Death Averted by Medicare at Age 65 for Our Tumor Sites of Interest

We calculated expenditure per cancer death averted by dividing the total Medicare expenditures associated with a breast, colorectal, and lung cancer diagnosis for 65‐year‐old patients by the number of breast, colorectal, and lung cancer deaths averted among 65‐year‐old patients. More precisely, we compared relevant changes in mortality at age 65 (i.e., nine cancer deaths avoided per 100,000 women at age 65, and no change among men) with related Medicare expenditures incurred at age 65 (i.e., Medicare expenditures associated with a cancer diagnosis for men and women with breast, colorectal, or lung cancer at age 65). We assumed that people diagnosed with breast, colorectal, and lung cancer at ages 60 to 64 who subsequently survived to age 65 would have their ongoing treatment costs at age 65 paid by Medicare.

To calculate the total costs to Medicare of covering cancer patients for care related to our tumor sites of interest, we used the following procedure. First, we calculated the number of people nationwide who become diagnosed with breast, colorectal, or lung cancer for each single year of age through age 65, by gender, by multiplying the detection rates in SEER by the total population nationwide for that age and gender. Second, we calculated the proportion of patients with each type of cancer, age, and gender who survive to age 65 and therefore become treated by Medicare at age 65. Third, we extracted the expected expenditures for Medicare associated with treating a patient with each type of cancer for 12 months, net of the expenditures for otherwise similar Medicare patients without that type of cancer, using estimates from the literature (Yabroff et al., 2008). We assumed that patients diagnosed at age 64 or 65 had incurred the expected expenditures that Yabroff et al. estimated for cancer patients in their first 12 months of treatment, whereas patients diagnosed at age 60 through 63 incurred the annual expected expenditures that Yabroff et al. estimated for cancer patients after their first year of treatment. We assumed that patients whose cancer was diagnosed prior to age 60 and survived to age 65 were in remission, and therefore incurred no additional expenditures at age 65 beyond those expenditures that would be incurred by patients without cancer. This assumption does not substantially change our findings.

To obtain the total expenditures related to cancer care to Medicare for our tumor sites of interest, we multiplied these three quantities together. In other words, we multiplied the number of patients diagnosed at each age, gender, and tumor site, by the probability that a patient diagnosed with cancer of that tumor site at that age and gender will survive until age 65, by the Medicare expenditures associated with treating their tumor at age 65 if they survive. Naturally, for patients diagnosed with cancer at age 65, the proportion surviving to age 65 was 1.

We observed a decline in cancer mortality of nine per 100,000 women at age 65, implying that 1,887 cancer deaths at age 65 were avoided over our time period of interest for our tumor sites of interest given the size of the population of women at age 65 nationwide during 2001 to 2015. We estimated that Medicare spent $9.5 billion treating cancer for our conditions of interest, given the size of the population of women and men aged 60 to 65 nationwide during 2001 to 2015 and the cancer diagnosis and survival rates at each of those ages. Dividing $9.5 billion spent by 1,887 cancer deaths averted yields an estimated expenditure of $5 million per cancer death averted at age 65.

**Table A1:** Changes in cancer detection and mortality at age 65 per 100,000 population by race and gender

A. All Tumor Locations								
	Black				Non‐Black			
	Women		Men		Women		Men	
	Age 63–64	RD at Age 65	Age 63–64	RD at Age 65	Age 63–64	RD at Age 65	Age 63–64	RD at Age 65
Cancer Detection								
Total	725.1	77.28***	465.9	9.600	678.8	70.87***	297	34.15***
		(28.21 to 126.3)		(−34.91 to 54.11)		(49.77 to 91.98)		(18.14 to 50.16)
Early‐Stage	398.6	61.74***	142.8	5.054	419.6	46.80***	96.86	17.85***
		(25.49 to 97.99)		(−22.09 to 32.20)		(31.06 to 62.53)		(8.82 to 26.90)
Late‐Stage	310.7	13.58	299.4	15.34	245.1	22.65***	186.8	16.27***
		(−16.66 to 43.81)		(−18.00 to 48.68)		(11.29 to 34.01)		(4.13 to 28.41)
Cancer Mortality	238.4	−20.29***	309.6	9.59	195.1	−3.32	208	0.38
		(−31.74 to −8.842)		(−6.65 to 25.83)		(−11.14 to 4.51)		(−7.90 to 8.66)
B. By Tumor Site								
Cancer Detection								
Total	725.1	77.28***	465.9	9.600	678.8	70.87***	297	34.15***
		(28.21 to 126.3)		(−34.91 to 54.11)		(49.77 to 91.98)		(18.14 to 50.16)
Breast Cancer	450.6	49.22***		N/A	460.2	51.21***		N/A
		(12.55 to 85.89)				(35.36 to 67.06)		
Colorectal Cancer	110.9	16.18	149.8	4.662	64.73	11.47***	91.12	18.00***
		(−3.239 to 35.60)		(−22.05 to 31.38)		(4.64 to 18.29)		(9.62 to 26.39)
Lung Cancer	163.6	12.05	309	4.601	153.9	6.927	202.6	15.97**
		(−13.38 to 37.47)		(−30.08 to 39.28)		(−3.774 to 17.63)		(2.37 to 29.57)
Cancer Mortality								
Total	238.4	−20.29***	309.6	9.59	195.1	−3.32	208	0.38
		(−31.74 to −8.84)		(−6.65 to 25.83)		(−11.14 to 4.51)		(−7.90 to 8.66)
Breast Cancer	80.97	−6.77*		N/A	57.39	1.63		N/A
		(−14.26 to 0.71)				(−1.05 to 4.30)		
Colorectal Cancer	46.01	−3.90	71.42	3.61	27.20	0.35	42.95	0.82
		(−8.64 to 0.84)		(−2.75 to 9.97)		(−1.41 to 2.11)		(−1.73 to 3.37)
Lung Cancer	111.5	−9.41*	237.1	4.67	110.5	−5.40	164.4	−0.50
		(−18.88 to 0.0677)		(−10.04 to 19.37)		(−12.14 to 1.34)		(−8.06 to 7.06)

*Notes*: This table shows changes in cancer detection and mortality at age 65. The rows include findings from models that only use participants in the gender and race group in the column title. Estimated regression discontinuities at age 65 adjust for background trends in aging and over time, and models of cancer detection additionally adjust for time‐invariant state‐level characteristics. **p*<0.1; ***p*<0.05; ****p*<0.01.

**Table A2:** Balance tests for social and economic characteristics at age 65 in the United States 2001 to 2015: BRFSS Data

	Age 63–64	RD at Age 65
Fraction Retired	0.43	0.012**
		(0.0003 to 0.025)
Fraction Currently Working	0.38	0.004
		(−0.007 to 0.014)
Fraction who are Veterans	0.19	−.010
		(−.0223 to .003)
Fraction with College Education	0.58	0.004
		(−0.008 to 0.016)

*Notes*: This table shows the results of regression discontinuity analyses of economic background factors at age 65 using the BRFSS data. Estimated regression discontinuities at age 65 are adjusted for background trends in aging and over time, time‐invariant confounders by state, and respondent gender and race. Models incorporated sample weights to account for the complex sampling design of the BRFSS data. 95 percent confidence intervals calculated using robust standard errors are in parentheses. **p*<0.1; ***p*<0.05; ****p*<0.01.

**Table A3:** Additional sensitivity analyses

A. Cancer Detection and Mortality Data		
	Cancer detection per 100,000 population per year		
	Total	Early‐stage	Cancer mortality per 100,000 population per year
Main Specification	50.05***	32.91***	−4.13
	(30.88 to 69.22)	(21.47 to 44.35)	(−14.31 to 6.05)
*Change Bandwidth*			
Larger Bandwidth (8)	45.88***	29.19***	−2.76
	(30.05 to 61.70)	(19.75 to 38.63)	(−11.59 to 6.06)
Smaller Bandwidth (4)	48.85***	25.01***	−1.90
	(21.02 to 76.67)	(10.44 to 39.58)	(−16.22 to 12.42)
*Change Order of Polynomial*			
Higher Order (3)	47.32**	27.74**	−4.20
	(10.99 to 83.64)	(6.289 to 49.19)	(−23.64 to 15.24)
Lower Order (1)	35.62***	24.61***	−0.40
	(24.53 to 46.71)	(18.01 to 31.21)	(−6.50 to 5.69)
Use Less‐Parametric Method	18.31**	12.87**	−5.36**
	(7.81 to 28.81)	(5.48 to 20.26)	(−2.67 to −8.04)

B. Cancer Mortality Data by Race and Gender				
	Black		Non‐Black	
	Women	Men	Women	Men
Main Specification	−20.29***	9.59	−3.32	0.38
	(−31.74 to −8.84)	(−6.647 to 25.83)	(−11.14 to 4.51)	(−7.90 to 8.66)
Larger Bandwidth (8)	−14.60***	6.86	−3.79	3.06
	(−24.54 to −4.65)	(−7.15 to 20.87)	(−11.09 to 3.51)	(−4.58 to 10.69)
Smaller Bandwidth (4)	−22.12***	13.22	−3.59	7.16
	(−37.02 to −7.21)	(−9.47 to 35.90)	(−14.22 to 7.03)	(−3.22 to 17.53)
Higher Order (3)	−28.10**	10.80	−4.63	7.41
	(−49.86 to −6.34)	(−21.51 to 43.10)	(−19.89 to 10.62)	(−7.61 to 22.43)
Lower Order (1)	−4.59	2.70	−1.46	1.97
	(−12.17 to 2.98)	(−8.29 to 13.68)	(−5.92 to 3.00)	(−3.01 to 6.95)
Use Less‐Parametric Method	−2.94**	−4.72**	−4.82**	−8.94**
	(−5.56 to −0.37)	(−8.73 to −0.72)	(−6.08 to −3.57)	(−10.61 to −7.27)

*Notes*: This table shows changes in cancer detection and mortality at age 65 under various model specifications, in the pooled data as well as by race and gender. The first row includes findings from the main specification, which used a bandwidth of 6 and adjusts for trends in age using a quadratic polynomial. In the subsequent rows, we change the bandwidth to 4 and 8 or adjust for aging effects using a more‐restrictive linear polynomial or less‐restrictive cubic polynomial. In the final row, we use a less‐parametric modeling approach, implementing a quadratic local polynomial estimator with robust bias‐corrected confidence intervals under second‐order Holder smoothness class using the *LPPHonest* function from the *RDHonest* package in R. This model used a triangular kernel to place a higher weight on observations closer to age 65. Exact *p*‐values are not available in the *RDHonest* package, and we therefore use stars to denote significance at the 0.05 level only. The release of the *RDHonest* R package available at the time of analysis did not accommodate covariates. Therefore, we incorporated covariates through a first‐stage partial regression process. The package additionally requires the user to specify an assumed bound of the second derivative of the conditional mean function. We chose this bound using the data‐based optimal selection procedure in the R package (*RD_MROT.fit*). In addition to the aging trends noted above, the models are adjusted for year and patient gender and race, and the cancer detection models are adjusted for state fixed effects. 95 percent confidence intervals are in parentheses. **p*<0.1; ***p*<0.05; ****p*<0.01.
